# CAR-T treatment for cancer: prospects and challenges

**DOI:** 10.3389/fonc.2023.1288383

**Published:** 2023-12-05

**Authors:** Ran Chen, Lei Chen, Chaoqun Wang, Hua Zhu, Lijuan Gu, Yuntao Li, Xiaoxing Xiong, Gang Chen, Zhihong Jian

**Affiliations:** ^1^ Department of Neurosurgery, Renmin Hospital of Wuhan University, Wuhan, China; ^2^ Cancer Center, Renmin Hospital of Wuhan University, Wuhan, China; ^3^ Central Laboratory, Renmin Hospital of Wuhan University, Wuhan, China

**Keywords:** CAR-T, cancer immunotherapy, tumor microenvironment, immunosuppressive environment, immune checkpoint

## Abstract

Chimeric antigen receptor (CAR-T) cell therapy has been widely used in hematological malignancies and has achieved remarkable results, but its long-term efficacy in solid tumors is greatly limited by factors such as the tumor microenvironment (TME). In this paper, we discuss the latest research and future views on CAR-T cell cancer immunotherapy, compare the different characteristics of traditional immunotherapy and CAR-T cell therapy, introduce the latest progress in CAR-T cell immunotherapy, and analyze the obstacles that hinder the efficacy of CAR-T cell therapy, including immunosuppressive factors, metabolic energy deficiency, and physical barriers. We then further discuss the latest therapeutic strategies to overcome these barriers, as well as management decisions regarding the possible safety issues of CAR-T cell therapy, to facilitate solutions to the limited use of CAR-T immunotherapy.

## 1 Introduction

Chimeric antigen receptor CAR-T cells can bind to recombinant chimeric tumor-associated antigen receptors. CAR-T cells often have costimulatory domains that function to enhance their activity, proliferation, and cytokine release (1–3). The various costimulatory molecules and components of CAR immune receptors help CAR-T cells recognize antigens and directly kill target cells (4–6). The US Food and Drug Administration (FDA) has recently approved several clinical trials, one of which has shown promising results in patients with relapsed B-cell malignancies or multiple myeloma. CD19 (CAR-T19) or B-Cell Maturation Antigen (CAR-T-BCMA) is the target of CAR-T tumor immunotherapy (7–10). However, some of the benefits achieved by CAR-T cell therapy are limited to patients with hematological malignancies. In contrast, the application of CAR-T cell immunotherapy in the treatment of solid tumors is challenging (11, 12).

Despite the high initial response rate to chimeric antigen receptor T cell immunotherapy, the high relapse rate for most patients cannot be ignored. Immunosuppression of the tumor microenvironment (TME), defective T-cell function, and tumor cell mutation may lead to resistance to CAR-T cell therapy. The TME is a complex collection of tumor cells, blood vessels, tumor-infiltrating immune cells, tumor-associated macrophages (TAMs), myeloid-derived suppressor cells (MDSCs), cancer-associated fibroblasts (CAFs), signaling molecules and extracellular matrix (ECM). These cells work together to change the TME into an immunosuppressive environment, inhibit the antitumor immune response, and promote tumor growth and metastasis (13). Tumor cells can resist the tumor-killing toxicity of CAR-T cells through the following: 1. Downregulation of target antigen expression and 2. Mutation of the death receptor. In addition, the early depletion of CAR-T cells may be related to the inherent defects of T cells themselves in patients with tumor diseases. Meanwhile, CAR-T cell activity was further reduced by the presence of immunosuppressive cells and factors, which are abundant in TME. In conclusion, unblocking TME in tumor tissue to antitumor immunotherapy will greatly promote long-term remission after CAR-T cell therapy. The exploration of various novel therapeutic strategies against CAR-T cell resistance provides a very important prerequisite for the success of early clinical trials (12).

In view of the antigenic heterogeneity and immunosuppressive environment of solid tumors, effective CAR-T cell therapy needs to be combined with strategies to recruit host immune responses to stimulate the recognition of tumor antigens that are not targeted by CAR-T cells and to reshape the immune cell composition of TME to support antitumor function. The energy metabolic requirements to better maintain the killing effect of CAR-T cells in TME will also improve the efficacy and durability of CAR-T cell therapy (14). A balanced cellular homeostasis mechanism promotes the survival and proliferation of cancer cells and enables them to evade immune surveillance by the body (15). The two-way interaction between cancer cells and the tumor microenvironment affects the occurrence and progression of cancer, further determining the therapeutic efficacy of tumors and the long-term prognosis of patients. Specifically, tumor cell-derived and remodeled highly interactively linked TME structures derive from the recruitment of cytokines and chemokines secreted by tumor cells, in which tumor stromal cells, immune cells and endothelial cells, chemotaxis aggregate and interact together to form a functionally intact tumor immune microenvironment (16). Studies have shown that for patients with solid tumors, tumor infiltration of CD8+ T cells leads to a better clinical prognosis. Various types of immune cells in the body can extensively infiltrate tumor tissue, and their differential phenotypes and compositions make their functions within the TME very different, leading to differential disease prognosis in patients (17, 18). In contrast, the presence of more M2-polarized macrophages or α-smooth muscle actin (SMA)-positive cancer-associated fibroblasts (CAFs) in the tumor microenvironment may predispose patients to a poor prognosis (19). Thus, the TME provides a wide range of potential therapeutic targets for improving the antitumor efficacy of CAR-T cells.

This review will introduce in detail the possible factors that limit traditional CAR-T immunotherapy, review the latest progress of CAR-T immunotherapy innovation and its progressiveness and challenges that are different from those of traditional cancer immunotherapy, and propose novel and promising immunotherapy strategies in the future to overcome these obstacles at the stage of preclinical research and development or clinical research.

## 2 CAR-T biological structure and function

The components consist of light/heavy chains, linkers, hinge regions, transmembrane regions, costimulatory molecules and stimulatory molecules. These proteins are distributed in the ectodomain, lipid bilayer and endodomain.

### 2.1 CAR-T biological structure

**Figure 1:**
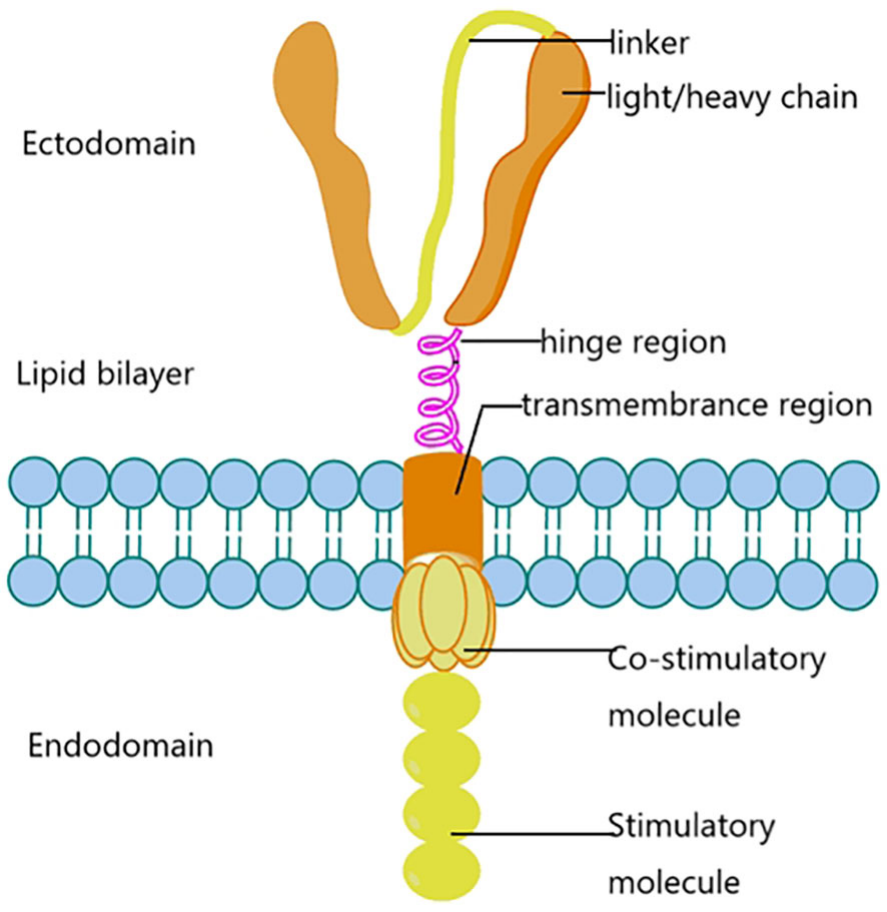
CAR-T biological structure.

CAR is composed of extracellular single-chain variable fragments, ScFv, transmembrane domain, immunoreceptor tyrosine-based activation motifs (Immunoreceptor tyrosine-based activation motifs), ITAM) and costimulatory signals of the cellular interior composed of synthetic receptors (20). ScFv is responsible for the recognition and binding of tumor-associated antigens (TAA) expressed on the surface of tumor cells (**Figure 1**). The transmembrane domain plays a key role in T cell activation, proliferation, persistence and cytotoxicity. The structure of CAR is similar to TCR, but its scFv recognizes TAA, does not depend on the majorhistocompatibility complex (MHC), and targets a variety of antigens expressed on the surface of tumor cells. Including proteins, carbohydrates, and gangliosides (21, 22). CAR-T cells recognize and bind TAA, induce conformational changes, and transduce binding signals into CAR-T cells, which are activated by activation signals in the CD3ζ domain and costimulatory domain, leading to cytokine release and transcription factor expression, promoting proliferation and persistent survival of T cells. And finally induced cytotoxic activity against tumor cells (23).

### 2.2 Evolution of CAR-T Cells

CAR-T therapy research has undergone five generations of development since the concept of CAR was first introduced. The concept of CAR was pioneered by Eshhar et al.The first generation of CAR is the basic structure of CAR-T, which typically consists of an antibody-derived extracellular antigen-binding structural domain, scFv, linked to an intracellular signaling structural domain containing the CD3ζ chain of the T-cell receptor (TCR) (24). However, first-generation CARs containing only CD3ζ sequences could not effectively activate CAR-T cells in the absence of co-stimulatory signals. In second-generation CAR-T, co-stimulatory structural domains fused with CD3ζ, such as CD28 or OX40, were added. Through the incorporation of co-stimulatory molecules, second-generation CAR-Ts showed optimized cell proliferation capacity, sustained response capacity, and cell killing toxicity (25). Third-generation CARs are designed by integrating an additional co-stimulatory structural domain in second-generation CARs, usually consisting of CD28/4-1BB/CD3ξ or CD28/OX40/CD3ξ in the intracellular structural domain. Early clinical trials in patients with leukemia and non-Hodgkin’s lymphoma have shown that third-generation CAR-Ts expand better and last longer (26, 27). Despite breakthroughs in the treatment of hematologic malignancies with second- and third-generation CAR-T cell therapies, their use in solid tumors remains limited. In addition, the occurrence of adverse events urgently calls for improvement in the safety of CAR-T immunotherapy. Fourth-generation CAR-T are designed to insert controlled switches, suicide genes, or elements that enhance T-cell function, such as caspase-9 gene elements or protease-based drug modulation platforms (28–30). The fifth generation CARs were designed as universal CARs containing either BBIR (biotin-binding immunoreceptor) or SUPRA. The BBIR CAR is a biotin-binding immunoreceptor consisting of an extracellular structural domain of affinities linked to the intracellular T cell signaling structural domains. The SUPRA CAR is a two-component receptor system consisting of a universal receptor (zipCAR) expressed on T cells and a separate scFv (zipFv) molecules that target specific antigens (31, 32). Universal CAR-T has the potential for large-scale production while reducing treatment costs. In summary, subtle changes in the structure of CARs from generation to generation have revolutionized CAR-T therapies and stirred great enthusiasm for cancer immunotherapy research.

## 3 Obstacles in CAR-T therapy in cancers

### 3.1 Immunosuppressive microenvironment

**Figure 2:**
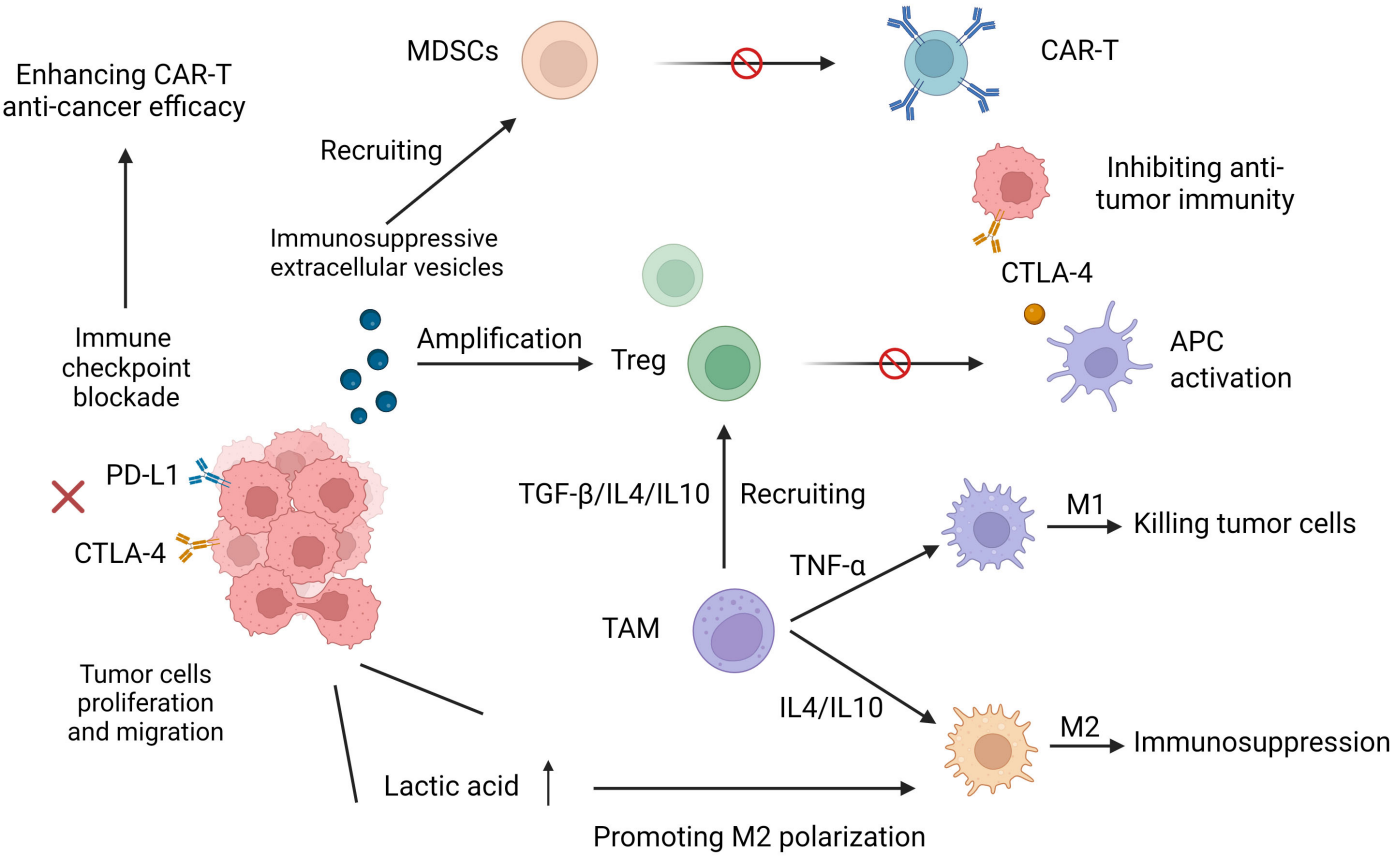
Increased expression of programmed cell death protein 1 (PD-1) and cytotoxic T lymphocyte-associated protein 4 (CTLA-4) reduces T-cell receptor (TCR) signaling sensitivity and leads to its dysfunction. Immunosuppressive cytokines and immunosuppressive cells (myeloid-derived suppressor MDSCs, tumor-associated macrophages (TAMs), and Treg cells hinder the effective antitumor function of CART cells. M1 phenotypic TAMs can phagocytose and kill tumor cells. M2 phenotypic TAMs put TME into an immunosuppressive state, which promotes tumor cell proliferation and distant migration and inhibits the killing effect of CAR-T on tumor cells.

Studies have shown that the immunosuppressive TME preferentially recruits Treg cells, MDSCs and tumor-associated macrophages (TAMs) to aggregate around the tumor, limiting the antitumor efficacy of CAR-T cells, in part because of the release of cytokines and chemokines. Different immunologically active forms of TAMs play very different opposing roles. M1 macrophages can phagocytose and kill tumor cells, exerting an antitumor effect on immune surveillance, a process that is detected in the initial stages of cancer development. However, in the middle and late stages of cancer development, the release of some cytokines (such as IL-4 and IL-10) leads to the polarization of TAMs to the M2 phenotype so that the TME enters an immunosuppressive state, thereby promoting tumor cell proliferation and distant migration and inhibiting the killing effect of T cells on tumor cells, affecting its antitumor effect (33). Treg cells can inhibit the functional activation of antigen presenting cells (APCs) mediated by cytotoxic T lymphocyte antigen 4 (CTLA4), partly through the competitive consumption of interleukin-2 (IL-2) by secreting immunosuppressive cytokines. Inactivation of APC inhibits T-cell activation and further inhibits cytotoxic T-cell function (34). MDSCs target effector T cells to exert a potent immunosuppressive effect while mediating the inhibition of CAR-T cell function. In a study of CAR-T19 treatment, lower levels of MDSCs in patients with lymphoma and leukemia gave them a more pronounced anticancer effect (26). TAMs in the TME are immune cells that significantly infiltrate and aggregate. In addition to regulating antitumor immune responses through phenotypic polarization, they can also promote the recruitment of Treg cells by secreting cytokines and amino acid-depleting enzymes to further inhibit T-cell-mediated antitumor responses and tumor immunity (35–37) (**Figure 2**).

In response to the immunoinflammatory suppressors in the immunosuppressive microenvironment described above, a study has developed a genetic platform binding the auxiliary molecules induced by autonomous antigens with constitutive CAR expression in the same lentiviral vector. Its function is threefold: (1) to increase the expression of immunostimulatory cytokines; (2) to ameliorate cytokine release syndrome; and (3) to modulate T-cell bioactivity. In conclusion, this study provides important research support for the transformation of CAR-T manipulation into more effective anti-tumor efficacy from the perspective of regulating immune cytokines, and it has important application prospects and research significance in the future to find out the design of more effective CAR-T strategies for solid tumors in the field of manipulating immunosuppressive cytokines (38).

Compared with virus antigen-specific T cells activated by acute infection, solid tumor autoantigen-specific functional T cells have the following characteristics. Solid tumor autoantigen-activated T cells face three obstacles. (I) Elimination of negative selection during immune maturation: fewer precursor cells leading to functionally mature tumor-specific T cells and only low to moderate affinity for TCRs (39). (II) Poor immunogenicity: Solid tumor cells are ineffective against costimulation. Thus, despite the increased number of tumor antigens and the progressive expansion of tumor cells, tumor-specific T cells are not sufficiently activated, resulting in a deficiency and limited function of effector and memory cells. In contrast, acute viral infection triggers highly immunogenic APCs, leading to potent T-cell activation and proliferation. (III) Extrinsic mechanism-mediated suppression of T-cell function: effector and memory T cells become unresponsive in the tumor microenvironment. Inappropriate TCR triggering, broad inhibitory receptor signaling, and other cytokines lead to functional deficits or early exhaustion of T cells and prevent normal differentiation of memory cells (40).

In addition, the elevated level of lactic acid, a high metabolic product of tumor cells, promotes the inhibition of T-cell signal transduction mediated by activated T-cell nuclear factor (NFAT) (41), Treg cell amplification (42), and macrophage differentiation into the immunosuppressive M2 phenotype (43). Other studies have shown that immunosuppressive extracellular vesicles, newly discovered extracellular vesicles secreted by tumor cells, also induce dysfunction of CAR-T cells (44).

In summary, the influential characteristics of the interaction between tumor cells, the TME and CAR-T cells suggest key obstacles to be overcome in the sustainability and efficacy optimization of tumor immunotherapy.

### 3.2 Inhibitory checkpoint receptor

**Figure 3:**
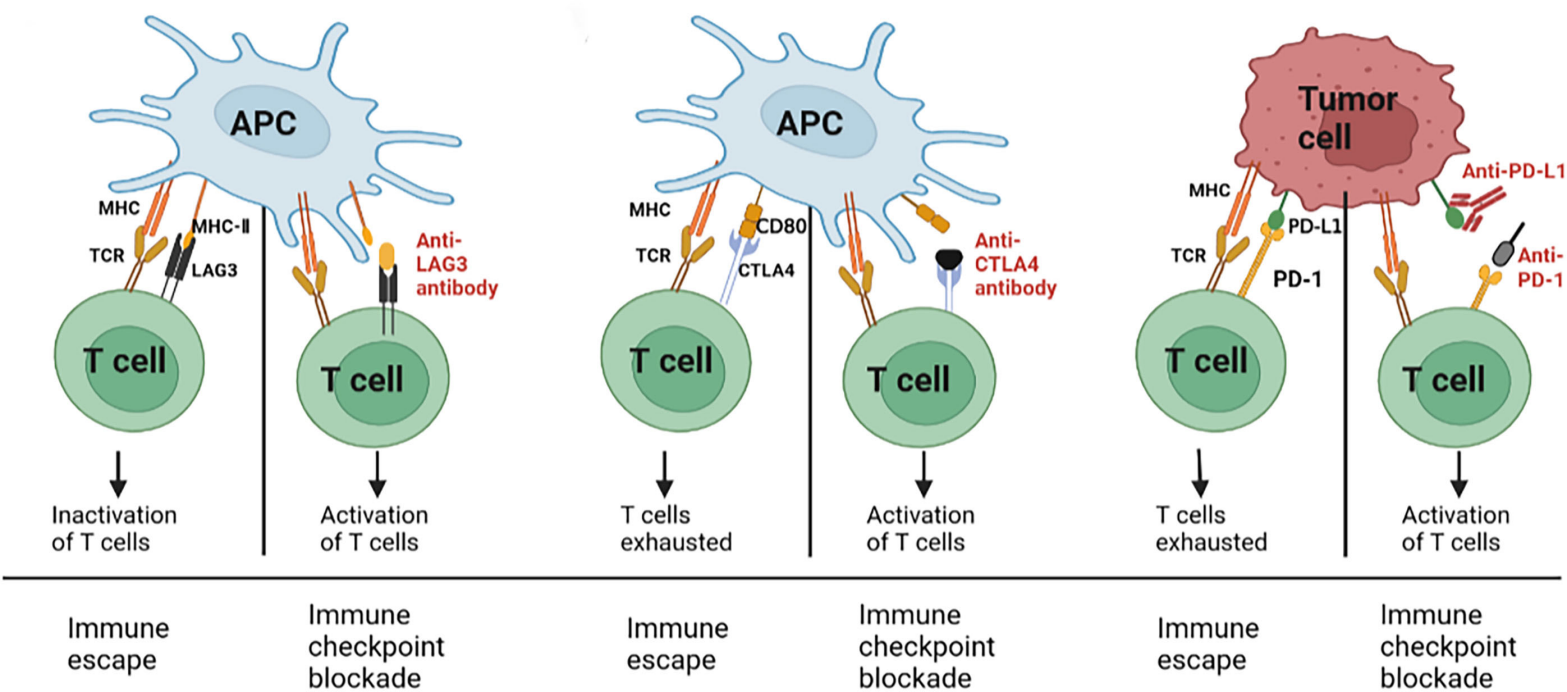
Immune checkpoint blockade of T-cell activation.

Many types of immune cells, such as T cells and NK cells, regulate the intensity and depth of the immune response by expressing inhibitory molecules called “immune checkpoint proteins”, which in turn regulate peripheral immune tolerance and prevent tissue damage caused by immune overactivation (45). Specifically, increased expression of programmed cell death protein 1 (PD-1), cytotoxic T lymphocyte-associated protein 4 (CTLA-4), and lymphocyte-activating gene 3 (Lag3) decrease TCR signaling sensitivity and inhibit its function (40). CTLA-4 is an inhibitory regulator of T lymphocytes. In mice, the loss of CTLA-4 leads to diffuse infiltration of lymphocytes, leading to immune hyperactivation. Conversely, inhibition of CTLA-4 activation was significantly beneficial in CAR-T immunotherapy of melanoma (46, 47). Another mechanism of tumor recurrence after CAR-T treatment is thought to be related to abnormal expression of PD-L1 on tumor cells, which mediates immunosuppressive signals, leading to early failure of CAR-T cells and impaired tumor killing function (48) (**Figure 3**). Therefore, after anti-PD-1 treatment, although no significant amplification of CAR-T19/20 cells is detected, circulating T cells are activated in the responders. Since PD-L1 expression has been detected in tumor cells of patients with relapsed/refractory diffuse large B-cell lymphoma (DLBCL) and pD-1 levels are elevated in tumor-infiltrating T cells, pD-1 blockade therapy in CAR-T cell therapy may improve the efficacy (49). Similarly, the dual blockade of LAG 3 and PD-1 during T-cell activation effectively enhances the proliferation and cytokine production of NY-ESO-1 (a “cancer-testicular” antigen that is highly expressed in epithelial ovarian cancer)-specific CD8+ T cells. These results suggest that the antitumor function of NY-ESO-1-specific CD8+ T cells can be potentially improved by targeting these inhibitory receptors (50). Therefore, targeting and regulating the activation of inhibitory checkpoint receptors will have a profound impact on improving the efficacy of CAR-T cell tumor immunotherapy.

Immune checkpoints, including PD-1 and CTLA-4, are expressed on activated T cells, and T cell activation is inhibited when activated T cells bind to ligands on tumor cells/antigen-presenting cells. These interactions can be blocked using monoclonal antibodies, which in turn activate T cells targeting tumor cells through the release of cytokines and cytotoxic particles from effector T cells, resulting in tumor killing.

### 3.3 Biomechanical barrier

Solid tumors also have TMEs similar to biophysical barriers that are not found in hematological malignancies, inhibiting tumor infiltration of effector T cells and CAR-T cells through various mechanisms, thereby impairing their antitumor efficacy. TGF-β acts directly on T cells by inhibiting the expression of chemokine receptors such as CXCR3, thereby limiting T-cell infiltration in solid tumors. Activation of TGF-β stimulates the production of extracellular matrix (ECM) proteins in stromal cells (such as CAFs), thereby forming a dense fibrotic TME, which in turn blocks the migration of T cells (51). Taken together, CAFs limit the cellular function of CAR-T cells in antitumor models through a complex and multifaceted mechanism of TGF-β-mediated cell-to-cell interactions (52).

Specific ECM structures in the TME composed of proteins (such as collagen, elastin, etc.) and glycoaminoglycans (GAGs) play an important role in the process of antitumor immunity. Collagen fibers assemble into fiber bundles and form elastic fibers in the TME upon the interaction of elastin and collagen fibers (53). The compact arrangement of stromal fibers in tumor tissue results in a narrow pore structure that is significantly different from the loose arrangement in healthy tissue (54). GAG is also involved in the formation of a dense TME. The deposition of GAG is involved in the formation of a dense tumor matrix through increased IFP (55). In addition, the narrow pore structure formed by the inherent vascular and lymphatic systems in TME tissues is closely related to the fibrous and porous structure also formed, which greatly restricts immune cell migration (56). The biophysical barrier formed by the cross-linking of the matrix fiber network in the ECM is one of the resistance mechanisms leading to the efficacy of immunotherapy, which causes the retention of CAR-T cells in the matrix around the tumor center and makes it difficult to form effective contact with tumor cells and interact with them to kill tumor cells (57). Pancreatic, breast and prostate cancers are the most common examples (58, 59).

Hypoxia in solid tumor tissues is often related to the composition of abnormal blood vessels. Hypoxic tissues secrete various chemokines to upregulate the expression of CTLA4 or LAG3 on Treg cells, as well as the PD-L1 ligand of MDSCs, TAMs and tumor cells, thus blocking the entry of antitumor T cells into the tumor core and promoting the recruitment of immunosuppressive cells (60). The expression of the adhesion molecules vascular cell adhesion protein 1 (VCAM1) and intercellular adhesion molecule 1 (ICAM1) is inhibited by dysfunction of the vascular system in tumor tissues, which inhibits the effective infiltration of T cells and magnifies the inhibitory effect (34, 60). Intravascular fluid shear stress mainly mediates the resistance effect of immunotherapy by weakening the immune surveillance of tumor cells and inhibiting the extravasation of T cells. Specifically, T-cell adhesion and extravasation are inhibited by blocking l-selectin interactions on the T-cell surface with ligands on endothelial cells. L-selectin, CD11b, and CD11a on neutrophils bind to tumor cell surface receptors to form a “protective shell” to evade immune surveillance under specific vascular shear stress (61, 62). In CAR-T immunotherapy of solid tumors, restricted migration, infiltration and phagocytosis are closely related to the physical stiffness of the matrix. Changes in its stromal tissue stiffness can also transform it to polarize to the M2 phenotype, which resides in the tumor immune microenvironment for a longer time, leading to the secretion of immunosuppressive factors (63). Part of the mechanism of immunotherapy resistance is closely related to the biophysical barrier of the TME. Therefore, regulating the TME to enhance the immune activity of tumor-associated T cells will become an important target for developing new therapeutic measures to overcome CAR-T cell resistance.

### 3.4 Differential interaction between T cells and tumor cells

Liquid and solid tumors interact with CAR-T cells in different ways. Thus, targeting the interaction sites between T cells and tumor cells may improve the therapeutic efficacy of solid tumors for CAR-T. The interferon gamma receptor signaling pathway is a relay station that mediates the interaction of CAR-T cells with tumor cells. Investigators performed a genome-wide CRISPR knockout screen for glioblastoma (poor CAR-T cell efficacy). The results showed that deletion of IFNγR1 in glioblastoma cells reduced the overall binding affinity of tumor cells to CAR-T cells. In contrast, deletion of the interferon gamma receptor signaling pathway did not make hematologic tumor cells less sensitive to CAR-T cells. Furthermore, differential expression of the cell adhesion pathway gene ICAM1 in solid tumors affects CAR-T tumor killing capacity. Specifically, the tumor-killing cytotoxicity of CAR-T is attenuated in antibody-blocked ICAM1 or ICAM1 knockout tumor models. In contrast, ICAM1 re-expression restored the tumor cell adhesion ability of CAR-T (64). The authors found that blocking IFNγR1 resulted in a loss of ICAM-1 (a ligand on CAR-T cells) signaling linked to IFNγR1 in tumors, inhibiting the adhesion of CAR-T cells in solid tumor tissue. In addition, IFNγ signaling was found to be obstructed in breast cancer cells, which have shown resistance to targeted T-cell therapy *in vitro (* 65). Therefore, the differences in IFNγR signaling among different tumor types may be of great significance for modifying and designing CAR-T cells or guiding drug combination therapy by optimizing T-cell-tumor interactions and further improving the efficacy of CAR-T therapy for solid tumors (66).

### 3.5 Rapid depletion of CAR-T cells

The tumor TME can induce upregulation of inhibitory receptors and an imbalance in cell metabolism, and the energy generated by glycolysis is not enough to ensure effective T-cell proliferation and activation, leading to the loss of CAR-T proliferation capacity and immune function. Such cells are named failed T cells. Depleted T cells produced during tumorigenesis or chronic infection are a special group of T cells with limited proliferation and differentiation and immune dysfunction. These cells have special differentiation programs and are specialized cell groups derived from memory precursor effector cells (MPECs) (67). T-cell depletion partly depends on the activation of trigger molecules such as PTPN2, IRF4 and Blimp-1, which continuously activate signal transduction of T cells through different pathways (68). In addition, Liu et al. found that IL-2 induces T-cell failure in the TME environment by activating STAT5; thus, cytokines also play an important role in T-cell failure (69). In addition, IL-10 and IL-35 released by Treg cells can also cause T-cell failure even though their functions overlap partially (70). However, the exhaustion of T cells greatly weakens tumor resistance. Therefore, understanding the mechanism of T-cell exhaustion is essential for antitumor immunity research (71). T-cell failure caused by these effects significantly reduces tumor resistance. Therefore, it is necessary to understand the specific mechanism of T-cell failure for antitumor immunotherapy research. Single-cell transcriptome analysis showed that genes involved in glycolysis were significantly upregulated and that the expression of transcriptional T cytokine 7 (TCF7) (T-cell self-renewal gene) was reduced in terminal failure T cells (72). T-cell glycolysis is the metabolism of glucose into lactic acid and the inhibition of pyruvate entry into mitochondria. This process is rapidly initiated by the T-cell receptor (TCR), which induces the activation of pyruvate dehydrogenase kinase 1 (PDHK1). This glycolysis process is necessary for the production of effector T-cell proinflammatory cytokines such as IFNγ to achieve immune function (73). The energy metabolism of actively proliferating cancer cells and CAR-T cells depends on glycolysis. Since tumor proliferation requires more nutrients than CAR-T cells, glycolysis of CAR-T cells is limited by excessive consumption of glucose by overactive cancer cells in the TME, which inhibits signal transduction between TCRs and CARs and ultimately reduces the efficacy of CAR-T cells by covering up the antitumor response (74–76). In addition, studies have shown that the upregulation of PD-1 signal transduction and mTOR activity can induce the early failure of T cells by promoting a series of metabolic changes, including mitochondrial respiratory inhibition, reduced glucose uptake and glycolysis disorder (77).

### 3.6 The manufacturing steps of therapeutic CAR-T cells are complicated

**Figure 4:**
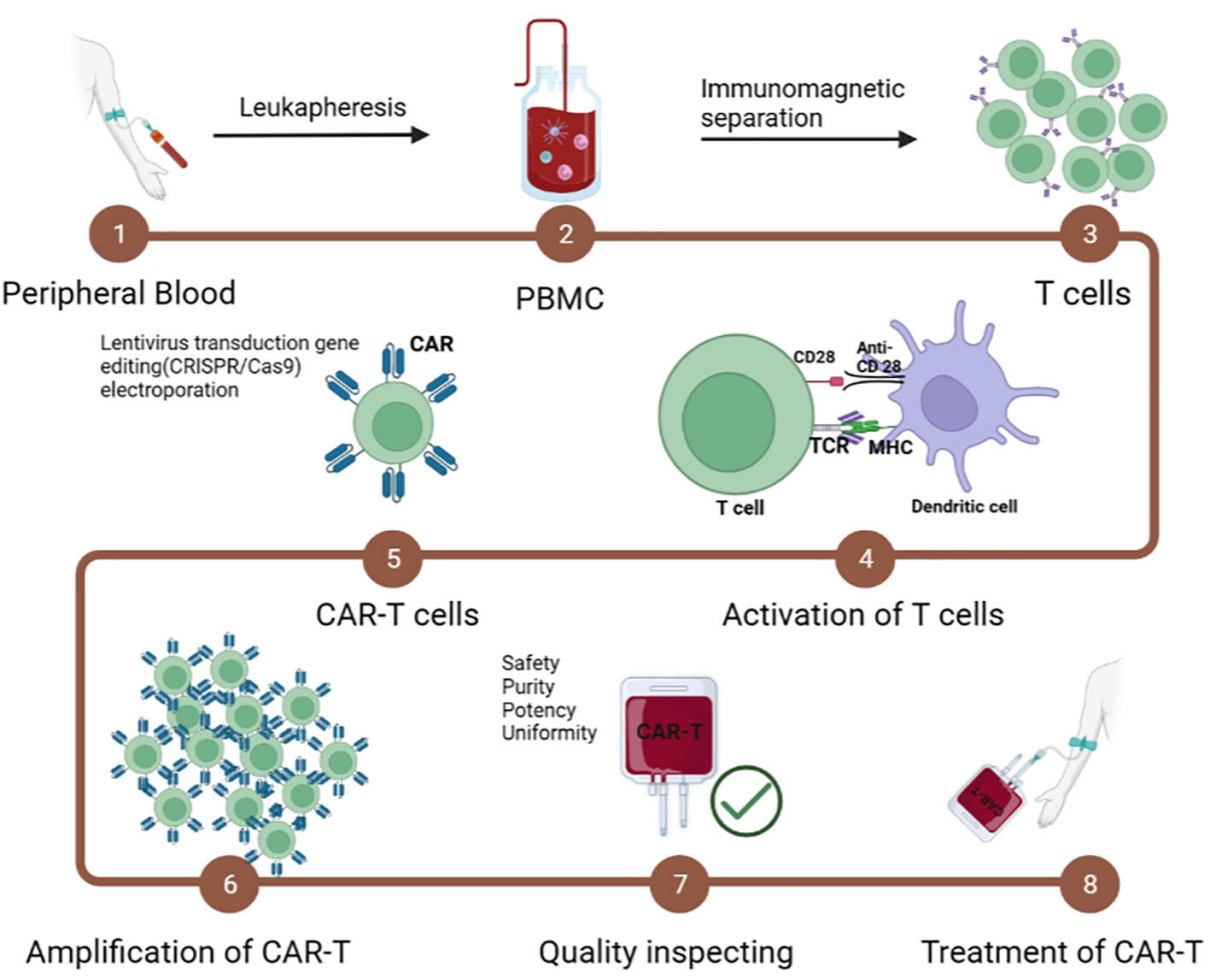
Manufacturing steps of CAR-T cells.

An important component of CAR-T treatment prep (lymphocyte clearance) promotes the biological function of effector T cells. Early studies have shown that lymphocyte clearance enhances the efficacy of sequential T-cell therapy (78, 79). Lymphocyte-depleting chemotherapy is now considered routine prior to CAR-T, with the combination of fludarabine (Flu) and cyclophosphamide (Cy) most often used (80, 81). The immunotherapy process of CAR-Ts in the human body is as follows: first, CAR-Ts are injected into the body and circulated in blood vessels, and then CAR-Ts are activated by ligands of tumor/malignant cells and proliferate/transform into memory CAR-Ts or produce a tumor killing effect in an appropriate microenvironment (**Figure 4**).

The immunotherapy process of CAR-T cells in the human body. The CAR-T cells are infused into the body and circulate in the blood vessels. The CAR-T cells are activated by the ligand of tumor/malignant cells and proliferate/transform into memory CAR-T cells or kill the tumor/malignant cell effector functions in the proper microenvironment.

Although CAR-T cell therapy is a promising immunotherapy for human malignancies, the high cost and complexity of CAR-T cell production are major obstacles to its eventual clinical use (82). Therapeutic CAR-T cells are expensive and time-consuming to produce. Many cancers are very aggressive, and most patients have a time gap waiting for the production of CAR-T cells to be completed. The specific steps include (1) isolation and collection of T cells; (2) transportation to the manufacturing center; (3) activation and amplification of T cells by virus vectors; (3) quality control; and (4) infusion of CAR-T cell products into patients (83–85). Therefore, the *in vitro* preparation of CAR-Ts has become a major limiting factor in clinical applications. In addition, T cells have undergone extensive proliferation and differentiation before entering the human body in advance in *in vitro* culture, resulting in limited activity and persistence of function of CAR-T cells *in vivo*, thus reducing their antitumor effect (86–88).

### 3.7 CAR-T cell therapy-related toxicity

#### 3.7.1 Cytokine release syndrome

CAR-T cells release many cytokines upon activation, such as GM-CSF, IL-6, IL-10, TNF-α and IFN-γ. In these cells, IL-6 levels were significantly higher compared to the levels of other cytokines. These cytokines therefore induce CRS. Lysed tumor cells release large amounts of cytokines, such as TNF-α. IFN-γ induces the activation of immune cells, especially macrophages. Activated macrophages release several cytokines, such as IL-6, IL-1, IL-10, TNF-α and NO. IL-6 can induce a strong immune response and plays a key role in the production of CRS. The release of a large number of cytokines, such as IL-6, TNF-α and IFN-γ, induces the activation of endothelial cells. Activated endothelial cells secrete large amounts of IL-6, which further promotes the pathological process of CRS and leads to cytokine storm. In addition to CRS, GM-CSF further exacerbates the neurotoxicity of CAR-T therapy by promoting the migration of inflammatory phagocytes to the central nervous system (CNS) and releasing reactive oxygen species and inflammatory cytokines. Therefore, GM-CSF is an important mediator for CRS and neurotoxicity in patients receiving CAR-T cell therapy. Research has proposed the development of GM-CSF−/− CAR-T cells (ablation of GM-CSF in CAR-T cells) as a potential strategy to reduce the CRS and neurotoxicity associated with CAR-T therapy (89).

CRS caused by rapid immune activation induced by CAR-Ts is the most important toxicity associated with treatment. CRS initially presents with fever and can progress to life-threatening capillary leakage with hypoxia and hypotension. Clinical signs of CRS are associated with T-cell activation and high levels of cytokines, including IL-6. The first clinical signs of CRS are fever, myalgia, and fatigue. Further exacerbation may lead to shock, vascular leakage, disseminated intravascular coagulation (DIC), and multiple organ dysfunction syndrome (MODS) (90). CRS can be self-limited (only supportive treatment with antipyretic drugs and intravenous fluids) or intervention with anti-cytokine therapy, such as corticosteroids or tocilizumab, may be required. CAR-T cell peak levels and serum IL-6 levels have been reported to be strongly and positively correlated with the severity of CRS after CAR-T cell therapy. For patients with severe CRS complicated by refractory hypotension and hypoxia, anti-IL-6 therapy with tocilizumab (IL6 receptor antagonist) or cetuximab is recommended (91). Tocilizumab or the chimeric anti-IL-6 monoclonal antibody cetuximab has become the drug of choice for the treatment of moderate to severe CRS (92). However, the use of tocilizumab in immune effector cell-associated neurotoxic syndromes (ICANS) is currently controversial, with some studies suggesting that tocilizumab treatment has a tendency to increase the incidence and severity of ICANS (93). Its exacerbation of ICANS may be due in part to the fact that tocilizumab is a monoclonal antibody that does not cross the blood-brain barrier, which blocks IL-6R in peripheral tissues and prevents systemic uptake of IL-20 (10). Thus, tocilizumab use may exacerbate the peripheral inflammatory response in ICANS.

CRS occur within 1 to 14 days after infusion, depending on the CAR-Ts product, clinical trial design, and treatment population. The duration of CRS is variable and complete remission usually occurs 2-3 weeks after CAR-T injection (94). Previous meta-analyses reported an approximately 55.3% incidence of CRS (95) and an approximately 18.5% incidence of severe cytokine release syndrome (96) in patients with hematological malignancies treated with CAR-T cells. In 2018, the American society for blood and bone marrow transplantation (ASBMT) pointed out that if the accept any immune therapy, the patient’s endogenous or inducing immune effector cells are activated in great quantities, so the resulting super physiological response must include the symptoms of fever, and may also include low blood pressure, blood capillary leakage (hypoxia) and end-organ dysfunction, This reaction is called CRS (97), which is the latest definition of CRS.

#### 3.7.2 Immune effector cell-associated neurotoxic syndromes

Immune effector cell-associated neurotoxic syndromes (ICANS), neurologic events occurring within the first few weeks of treatment, are considered to be a serious adverse effect of CAR-T therapy, with observed events including encephalopathy, delirium, aphasia, focal dysfunction, and seizures (9, 98). The pathogenesis of neurotoxicity is not well understood but may be mediated by the spread of cytokines in the super physiological state into the central nervous system (CNS) and/or direct T-cell infiltration. High levels of IL-6 and TNF-α on day 1 after CAR-T infusion were independently associated with the development of grade 4 CNS toxicity (99). Anti-CD19 CAR-Ts have been established to readily cross the BBB and can be detected in cerebrospinal fluid in most treated patients (92, 100). Studies have reported that the pathogenesis of ICANS may be related to the following factors: (a) the increased levels of IL-1, IL-6, IL-15, TNF-α and IFN-γ in the blood, which are positively correlated with the severity of ICANS (101). These cytokines promote the development and progression of ICANS; (b) Activation of endothelial cells in the central nervous system (CNS) leads to increased permeability of the blood-brain barrier (BBB), which allows cytokines from the blood to enter the cerebrospinal fluid (CSF) and promotes the development of ICANS (102, 103). Unlike fever and hypotension caused by systemic CRS, neurotoxicity does not respond easily to tocilizumab. Although corticosteroids are the mainstay of treatment, their effects on neurotoxicity have not been clearly defined (99, 104).

#### 3.7.3 Tumor inflammation-associated neurotoxicity

Another toxicity syndrome that differs from CRS and ICANS in the toxic effects of CAR-T cell therapy is termed tumor inflammation-associated neurotoxicity (TIAN), in which the tumor-associated inflammatory neurotoxicity is localized rather than systemic. TIAN is thought to be secondary to the inflammatory and targeting effects of CNS tumors, and the specific symptoms of TIAN reflect the effects of edema due to local inflammation. Edema associated with TIAN can cause tissue displacement, impede cerebrospinal fluid (CSF) flow, increase intracranial pressure, and may even lead to brain herniation. According to the different mechanisms by which neurotoxicity occurs, some studies have classified TIAN into two types: type 1 TIAN mainly reflects inflammatory edema leading to increased intracranial pressure and mechanical space limitation. type 2 TIAN mainly reflects the presence of specific neurological dysfunction caused by an immunotherapy-associated localized inflammatory response, in which inflammatory signaling molecules affect electrophysiological dysfunction within neural circuits (105). It has been shown that GD2 CAR-T cell therapy can effectively eliminate tumors in mouse models with H3K27M mutations in the thalamus and spinal cord. During the treatment, some mice developed peritumoral brainstem inflammation, compression of the fourth ventricle, obstructive hydrocephalus, and even a small percentage of mice died (106). Similar preclinical studies provide an important research basis for the future development of novel therapeutics and overcoming the therapeutic toxic effects of TIAN in CAR-T immune cell therapy.

## 4 Latest progress in CAR-T treatment

### 4.1 Overcoming the inhibitory effect of the TME on CAR-T antitumor cells

Significant concentrations of cancer-associated fibroblasts (CAFs) in the inhibitory TME of multiple myeloma (MM) in patients with relapsed/refractory MM have been shown to inhibit the antitumor activity of CAR-T cells and promote MM progression. However, B-cell mature antigen (BCMA)-targeted chimeric antigen receptor CAR-T cell therapy has yielded significant results in MM patients. Nonetheless, depletion of CAR-T cells and inhibition of the tumor microenvironment (TME) has led to insufficient persistence of efficacy. The dual targeting of CAR-T cells to MM cells and CAFs has overcome the CAR-T cell inhibition induced by CAFs and significantly improved the antitumor effect of CAR-T cells to MM cells (107).

CAR-T cells expressing multipotent proinflammatory neutrophil activator protein (NAP) (from Helicobacter pylori) have been shown to trigger endogenous T-cell responses to solid tumors, improving therapeutic outcomes against solid tumors. It has been demonstrated that CAR-T cells expressing bacteria-derived virulence factors can mediate the activation of endogenous immune cells, such as neutrophils, DCs and monocytes, to enhance stronger antitumor immunity and reverse the inhibitory immune microenvironment in solid tumors, thus achieving enhanced antitumor efficacy (108).

CAR-T cells interact differently with blood system cancer cells than with solid tumor cells, and enhancing the binding interaction between T cells and tumor cells may produce a better response in solid tumors (64). Studies have shown that IFN-γ signaling enhances CAR-T cell activity by overcoming PD-L1/PD-1 inhibition, significantly enhancing the antitumor activity of CAR-T cells *in vitro* and *in vivo*. Moreover, one study showed that the inhibitory function of PD-L1/PD-1 on CAR-T cell activity depended on IFN-γ signal silencing in tumor cells (66). In addition, recently developed GD2-directed chimeric antigen receptor CAR-T cells have demonstrated good preclinical efficacy in the treatment of solid tumor H3K27M mutated diffuse midline glioma. And this new application has entered clinical trials, with initial good results in patients (109).

Dense and disordered collagen in extracellular matrix (ECM) collagen is one of the main factors of abnormal structure in tumor ECM. Collagen content in the TME can be reduced by inhibiting enzymes that regulate collagen synthesis and secretion. CAFs can also be directly targeted for collagen production. Both approaches increase the tumor bed infiltration of T cells, thereby reducing immunotherapy resistance. In addition, the cross-linking of collagen and the elastin network mediated by lysyl oxidase (LOX) is essential for the structural maturation of the ECM, so inhibition of LOX activity improves the immunosuppression of the TME. For example, the use of LOX inhibitors in a mouse breast cancer model resulted in a more uniform distribution of the drug in the tumor and an increase in the total number of infiltrations, increasing the sensitivity of the tumor tissue to doxorubicin (110). Similarly, collagenase treatment of human lung tumor slices reduced stromal collagen content and significantly increased the number of infiltrated T cells in the tumor core (58). Direct degradation of the ECM, such as the development of novel nanomaterials, may also eliminate the immune blocking effect of the ECM, thereby enhancing emerging therapeutic strategies for immune infiltration. For example, in pancreatic cancer, collagenase nanoparticles were used to significantly reduce collagen levels in tumor ECM, improving the uptake rate of chemotherapy drugs (111).

Since cancer-associated fibroblasts are key members of the ECM composition, inhibition of fibroblast recruitment, activation and function is expected to improve the structure of the tumor ECM. It is known that fibroblast activated protein (FAP) is overexpressed on the surface of CAFs. Some studies have targeted FAP on the surface of CAFs with nanodelivery tools, enabling it to specifically locate CAFs, successfully reducing the aggregation of ECM, promoting tumor infiltration of T cells, and reversing immunosuppression (112). In another study that specifically improved the tumor microenvironment, poly(lactic acid glycolic acid) nanoparticles coated with indocyanine green were injected into tumor tissues of melanoma model mice in combination with near-infrared irradiation. The combination therapy inhibited ECM production and increased CAR-T cell invasion in tumor tissue compared with CAR-T cell therapy alone (113). The application of chimeric antigen receptor T cells (CAR-T cells) in solid tumors is the current development trend of tumor-based immunotherapy. In basic research, the combination of nanomaterials and CAR-T immunotherapy for ECM degradation has been developed successively, with the hope to further improve or even reverse the immunosuppressive state of the tumor microenvironment.

Dual targeting of CAR-T cells to tumor cells and CAFs can simultaneously inhibit ECM production and kill tumor cells. NAP-expressing CAR-T cells mediate the activation of endogenous immune cells (neutrophils, DCs and monocytes), reversing the suppressive immune microenvironment in solid tumors and enhancing antitumor efficacy. Activated IFN-γ signaling overcomes the inhibitory effect of PD-L1/PD-1 on CAR-T cell activity and significantly enhances the antitumor activity of CAR-T cells.

### 4.2 Enhanced CAR-T cell proliferation and antitumor effect

At present, the limitations of using chimeric antigen receptor T (CAR-T) cells to treat patients with blood cancer include limited *in vivo* expansion and persistence, which easily leads to cancer recurrence. A new study recently showed that CAR-T cells made with duvelisib (DUV CAR-T cells) showed a significant increase in cytotoxicity to CD19 + CLL (chronic lymphocytic leukemia) targets *in vitro*, a significant increase in the expansion of CD8 + CAR-T cells *in vivo*, and reduced expression of depletion markers in CAR-T cell products. They had stem cell-like characteristics, and CLL elimination was faster and longer, which enhanced the efficacy of eliminating CLL *in vivo (* 114).

Another study developed a CAR-T delivery method that promotes the proliferation of CAR-T cells and the production of stimulating cytokines by using polymer-nanoparticle hydrogel technology, thus generating a local immune microenvironment with improved antitumor efficacy (115). Therefore, the massive expansion of more tumor-responsive CAR-T cells *in vivo* greatly reduced the effective therapeutic dose of CAR-T cells, thus reducing the cost of treatment and hopefully providing new therapeutic strategies for refractory solid tumors.

In addition, the antitumor function of T cells can be regulated by metabolic reprogramming. Proline and arginine metabolism are known to be important in T-cell antitumor activity (116). Encoding proline dehydrogenase 2 into T cells can promote T-cell proliferation, activation and immune function. Total transcriptome analysis, multiomics analysis, cellular and immunological assays, and metabolic analysis combined with dgRNA and functional gain reprogrammed T-cell metabolism show that proline metabolism can be modified to improve the long-term efficacy of cancer treatment (117).

At present, the production process of autologous CAR-T first requires individualized blood separation and manufacturing (118), which increases the cost and effective dose of clinical application of CAR-T, reduces the production efficiency and *in vivo* activity of T cells, and becomes a major obstacle on the road of antitumor immunotherapy (119). In *in vitro* organoid culture, CAR iPSCs were differentiated into highly functional CAR-T cells. Specifically, iPSC CD19-CAR-T cells with a canonical T-cell phenotype were produced using genetic modification, and tumor cell lethality and cytokine secretion activity comparable to CAR-T cells obtained by conventional production methods were obtained. Therefore, genetic engineering technology can improve the efficiency of producing therapeutic CAR-T cell products by using iPSCs and reduce the production cost (120). Another study described a multifunctional alginate vector that can load host T cells and virus particles, stimulate T-cell activation and locally amplify CAR-T cells, release effector CAR-T cells and simplify the manufacturing process of CAR-T cells. This multifunctional scaffold mediates the release of fully functional CAR-T cells in mice to control tumor growth in a xenograft model of mouse lymphoma. Therefore, by transplanting the CAR-T cell production process into the body, then into the blood and controlling tumor proliferation, we achieved a longer and more powerful therapeutic effect than traditional CAR-T cells (121).

### 4.3 Reducing the toxicity of CAR-T

Fasting and drinking water, nutritional support therapy, and modified neurologic examination were performed for grade 1 ICANS, while either toibizumab (8mg/kg, intravenous > 1 hour) or metuximab (11mg/kg, intravenous > 1 hour) was performed. Intravenous administration > 1 hour) can be used in combination with grade 1 ICANS and CRS. In addition, for grade 2 ICANS, tocilizumab (8mg/kg, administered intravenously for >1 hour) or siltuximab (11mg/kg, administered intravenously for >1 hour) was administered. Glucocorticoids (dexamethasone 10mg/kg/6h or methylprednisolone 1mg/kg/12h) should be administered if the above drugs are ineffective or ineffective, or if comorbidities of ICANS and CRS have been reported. For grade 3 ICANS, it is recommended to transfer the patient to the ICU for further treatment and to administer glucocorticoids (at the same dose as above until the patient improves to grade 1 ICANS, and then gradually reduce the dose). Patients with grade 4 ICANS were given high-dose glucocorticoids (methylprednisolone 1g/day for 3 days, followed by gradual dose reduction; The entire treatment lasted 9 days (91).

## 5 Conclusions

Although CAR-T cell therapy is effective in the treatment of hematological malignancies, existing studies have shown that CAR-T therapy is not ideal for the treatment of solid tumors and their long-term prognosis. In addition, the extensive clinical application of CAR-T cells requires large-scale production, but the problems of time-consuming production and preparation of products and difficult storage and transportation are difficult to overcome with existing technologies. Recent studies have revealed many possible mechanisms that may hinder the efficacy of CAR-T cell therapy.

Further exploration of the internal mechanisms in the TME-related immunosuppressive environment, competition for energy metabolism substrates, and regulation of CAR-T proliferation and immune activity will facilitate the development of new and more efficient CAR-T products. These strategies include the use of novel T-cell metabolic reprogramming and gene editing methods that not only promote the delivery of more CAR-T cells to the tumor core and prevent the suppressive effect of the TME on CAR-T cells but also effectively activate the host innate immune response to synergistically enhance antitumor tumor effects. In addition, simultaneous targeting of more than one antigen is an important application of biological logic gating in CAR-T cells as a potential remedy to prevent antigen escape (122). Thus, future studies will focus on editing the application of bio-logic gating to CAR-T cells to make them more immunoselective and safe. Ultimately overcoming the hurdles of the TME in CAR-T cell therapy is a complex and arduous task and will require a multidisciplinary approach that acts synergistically with other treatment modalities to advance the development of new treatments for refractory human malignancies and strategies to achieve durable clinical benefit and maximize patient survival.

## Author contributions

RC: Writing – original draft. LC: Writing – original draft. CW: Writing – original draft. HZ: Writing – original draft. LG: Writing – review & editing. YL: Conceptualization, Writing – original draft. XX: Writing – review & editing. GC: Writing – review & editing. ZJ: Writing – review & editing.
